# Pepblock Builder VR – An Open-Source Tool for Gaming-Based Bio-Edutainment in Interactive Protein Design

**DOI:** 10.3389/fbioe.2021.674211

**Published:** 2021-05-14

**Authors:** Venkata V. B. Yallapragada, Tianshu Xu, Sidney P. Walker, Sabin Tabirca, Mark Tangney

**Affiliations:** ^1^Cancer Research @ UCC, University College Cork, Cork, Ireland; ^2^SynBioCentre, University College Cork, Cork, Ireland; ^3^School of Computer Science and Information Technology, University College Cork, Cork, Ireland; ^4^Department of Computer Science, Transylvania University of Braşov, Braşov, Romania; ^5^APC Microbiome Ireland, University College Cork, Cork, Ireland; ^6^iEd Hub, University College Cork, Cork, Ireland

**Keywords:** virtual reality, protein gaming, molecular visualisation, edutainment, 3D structure

## Abstract

Proteins mediate and perform various fundamental functions of life. This versatility of protein function is an attribute of its 3D structure. In recent years, our understanding of protein 3D structure has been complemented with advances in computational and mathematical tools for protein modelling and protein design. 3D molecular visualisation is an essential part in every protein design and protein modelling workflow. Over the years, stand-alone and web-based molecular visualisation tools have been used to emulate three-dimensional view on computers. The advent of virtual reality provided the scope for immersive control of molecular visualisation. While these technologies have significantly improved our insights into protein modelling, designing new proteins with a defined function remains a complicated process. Current tools to design proteins lack user-interactivity and demand high computational skills. In this work, we present the Pepblock Builder VR, a gaming-based molecular visualisation tool for bio-edutainment and understanding protein design. Simulating the concepts of protein design and incorporating gaming principles into molecular visualisation promotes effective game-based learning. Unlike traditional sequence-based protein design and fragment-based stitching, the Pepblock Builder VR provides a building block style environment for complex structure building. This provides users a unique visual structure building experience. Furthermore, the inclusion of virtual reality to the Pepblock Builder VR brings immersive learning and provides users with “being there” experience in protein visualisation. The Pepblock Builder VR works both as a stand-alone and VR-based application, and with a gamified user interface, the Pepblock Builder VR aims to expand the horizons of scientific data generation to the masses.

## Introduction

Proteins mediate and perform various fundamental functions of life. This versatility of protein function is an attribute of its 3D structure. Understanding the protein 3D structure is crucial for various fields of science. Elucidating the structure of a protein revolutionised the field of protein science and paved the way for the establishment of massive databases. Traditionally, physical methods such as NMR spectroscopy and X-ray crystallography have been deployed to elucidate and study the 3D structure of proteins. The recent advances in computational sciences have resulted in sophisticated algorithms for predicting and modelling the 3D structure of a protein from its corresponding amino acid sequence. Designing entirely novel proteins with a defined structure is also feasible with the current developments in *de novo* protein design (Huang et al., 2016). Traditionally, proteins were primarily designed by sequence-based design. In recent years, other concepts such as parametric modelling (Wood et al., 2017) and fragment assembly (Huang et al., 2011) have been developed to effectively design protein structures with particular functions. In spite of the recent progress, the field of protein design has a steep learning curve and requires great technical skills to effectively design novel protein structures. This lack of a complete visual-based interactive design tool is one of the bottlenecks in translating the protein design technology toward a broader creative audience. Recently (Yeh et al., 2018), Yeh et al. introduced a building block style graphical interface Elfin UI, which provides a modular approach to structure building. Such visual-based interactive interfaces create a platform for users with limited technical knowledge to create novel protein structures with minimal/no focus on the amino acid sequence.

### Visualising Proteins in 3D and Why?

**FIGURE 1:**
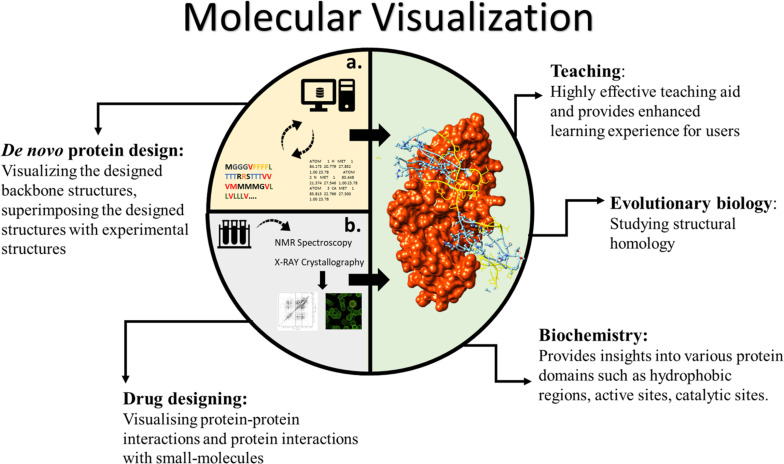
Various applications of molecular visualisation. The interior graphics refer to **(A)** connection between computational “protein modelling and design” and visualisation. **(B)** Showing the connection between physical structure elucidation methods and visualisation.

Visualising proteins in 3D has various unique applications and advantages for both scientific and educational purposes (Figure 1). In education, teaching the protein structure is a highly complicated task (Richardson and Richardson, 2002). The differences between the primary, secondary, and tertiary structures of proteins are challenging to understand. The non-linearity in protein folding, stereo-configurations and formation of large complex assemblies add to the existing hurdles in teaching the protein structure. In such cases, deploying computational 3D visualisation tools to visualise proteins not only provides users with an enhanced visual experience but also increases interest in the scientific field (Cai et al., 2021).

From a scientific perspective, visualisation of the 3D structure of a protein forms a pivotal component in both modelling the protein structure (physical methods and/or computational prediction) and *de novo* protein design. (i) *For biochemists:* It provides insights into various protein domains such as hydrophobic regions, active sites, catalytic sites etc. (ii) *For evolutionary biologists:* Visualising and mapping 3D structures aids in studying homology in structure. (iii) *In drug designing:* Visualising protein–protein interactions and protein interactions with small molecules can be key to understanding drug activity. (iv) *In de novo protein design:* Visualising the designed backbone structures and superimposing the designed structures with experimental structures is very commonly performed using *in silico* molecular visualisation.

### Current Tools for Visualising Proteins

Over the years, a wide variety of visualisation tools have been developed and deployed for 3D structure visualisation. These tools range from printout stereoscopic images to sophisticated VR CAVEs. Early 21st century saw a sudden increase in the number of protein structures deposited in protein databases (Bank, 2021), and a need for better visualisation tools increased in parallel. PyMOL (2021), VMD (Humphrey et al., 1996), Chimaera (Pettersen et al., 2004) and Rasmol (Sayle and Milner-White, 1995) are some examples of widely used stand-alone applications for molecular visualisation. Later, web-based applications such as Jmol (2021) and iView (Li et al., 2014) have gained interest in the scientific community. The web-based applications provided a way to integrate the visualisation into websites. Recently, several mobile-based applications for Android and iOS have also been developed by various groups for molecular visualisation. Although these tools provide a wide canvas of features for visualising proteins in 3D, there are a few considerable drawbacks such as the lack of full visual immersion and the lack of a real 3D effect.

Virtual reality (VR) based methods provide an alternative solution for these problems (Goddard et al., 2018a). Since its invention in the late 1960s, a wide range of technologies have been researched and deployed for VR (Indhumathi et al., 2007). Head mount devices (HMDs) such as Oculus Rift, Cave automatic virtual environment (CAVEs) and smartphone-based Google cardboard-like devices are some well-known examples. The introduction of VR brings a full immersive visual experience to molecular visualisation. The 3D effect generated in virtual reality provides other advantages such as enhanced accuracy in colour depiction of the models, accurate depth perception, improved wide field of view, haptic interfacing and better molecular viewing resolution (Norrby et al., 2015).

Visualising biomolecules (proteins in particular) in VR has gained wide attention recently (Goddard et al., 2018a). Tools such as ChimeraX (Goddard et al., 2018b), BioVR (Zhang et al., 2019), and StarCave (DeFanti et al., 2009) (cave based) have been developed for visualising the 3D structure of proteins in VR. Although the current technology provides a plethora of functionalities for the user, the potential of molecular visualisation in VR is still a maturing field. Easier navigation in the VR environment, better user interface (UI), faster rendering and simplified instrumentation are some areas that are expected to see some improvements in the near future. Parallel advancements in affordable VR headsets and increasing computational power and graphics project interesting times ahead.

### Gamification in Education and Scientific Research

Computer games are powerful audio-visual teaching tools and have been used as interactive learning aids in various fields of education. Gamification of scientific learning is becoming a popular form of edutainment. Today, edutainment is a powerful form of experiential smart learning (Anikina and Yakimenko, 2015), and the market value of edutainment is projected to reach 11.34 billion by 2028 (Global Edutainment Market Growth, 2021). Through fun-based learning, gamification instils curiosity, motivated experience and interest in learning (Cai et al., 2006, 2008). With the advances in human–computer interaction strategies, gamification has also expanded the realms of research by taking advantage of the massive number of citizen scientists to contribute toward complicated scientific goals. When combined with audio-visual edutainment and gamification, scientific goals appeal to a larger audience. The path from concept building, world realisation and problem solving are an integral part of gaming. This path is commonly observed in real-life scenarios such as scientific research. By mimicking scientific research and by adding gamification principles, tools/games for bio-edutainment such as fold.it and Phylo have gained public attention and resulted in remarkable scientific achievements (Koepnick et al., 2019) (Table 1).

**TABLE 1:** Examples of popular games for science and bio-edutainment.

Game/Tool	Scientific problem addressed	Description
Foldit (Kleffner et al., 2017)	Protein folding	Game involving real-time manipulation of protein structures, with results used to solve real-life problems. Recently resolved the structure of HIV-associated enzyme.
EteRNA (Anderson-Lee et al., 2016)	RNA folding	Puzzle-based game to provide insights into RNA design.
The Cure (Good et al., 2014)	Phenotyping in breast cancer	Detection of molecular signatures linked to specific breast cancer prognosis through web-based game.
Phylo (Kwak et al., 2013)	DNA sequence alignment	Web-based Tetris style game facilitating sequence alignments.
EyeWire (Cooper et al., 2018)	Neuronal mapping	Web-based 3D neuron reconstruction game.
Brainflight (Brainflight, 2021)	Neuronal mapping	Game to track the path of electric impulses travelling through the brain.

### Introducing Pepblock Builder VR

The Pepblock Builder VR is a gamified protein visualisation and design tool for bio-edutainment and fully visual-based protein structure building. The Pepblock Builder VR is currently available in two versions: (i) a stand-alone desktop-based tool and (ii) an HMD-based tool in the VR environment. The Pepblock Builder VR is a versatile tool that can serve as a visualisation tool, an edutainment tool and a structure-based modular design tool. As a visualisation tool, the Pepblock Builder provides an immersive experience to the users to visualise complicated 3D structures. As an edutainment tool, the Pepblock Builder VR provides an interactive and experiential learning experience to users through gamification. As a design tool, the modular (semi-LEGO^®^ style) protein design approach of the Pepblock Builder VR offers a multitude of non-technical users to build complex protein structures.

## Materials and Methods

### Ethics

Written informed consent was obtained from the [individual(s) and/or minor(s)’ legal guardian/next of kin] for the publication of any potentially identifiable images or data included in this article.

### PC Infrastructure Used for Pepblock Builder VR

The Pepblock Builder VR was developed on a desktop PC running Windows 10, with 8 GB RAM, 4GB NVidia graphics card and Intel core I7 processor (7th generation). Two 16-inch LCD monitor screens were used for display. Standard keyboard and optical mouse were used as input devices. The development of the Pepblock Builder VR demanded a high-spec configuration to facilitate multitasking during the design, implementation and testing stages of various iterations of the software in parallel (Table 2).

**TABLE 2:** Key features of Pepblock Builder VR.

Technical Features	Details/Format
File types	PDB, X3D
Structure visualisation format	Ribbons and Cartoons
VR module	Oculus Rift
Human interaction in VR and PC	Handheld controllers and optical mouse
Manipulation features	Rotation, zooming, bending and twisting carbon alpha chains, 360 X, Y, Z movements
GUI model and scheme	Space neon colour scheme
Graphics and Game mechanics tools used	Blender and Unity game engine

### VR Setup

Oculus Rift and Oculus Rift S setups were used for the development of the Pepblock Builder VR for virtual reality environment. The setup included an HMD, two handheld controllers for human–VR interaction and two stand sensors. The program has a pre-built library called OVR Utilities Plugin (Oculus Integration) for implementing functionalities with Oculus Rift. This enables the connection of external parts such as controllers, headset etc., to the VR environment.

### Data Generation, Protein Modelling and *in silico* Parameters

Protein tertiary structures were generated by the I-TASSER suite (v5.1). C-scores for all the structures were obtained from I-Tasser. *In silico* parameters such as hydrophobicity and theoretical pI were calculated using the ProtParam facility, hosted by Expasy. Saves server (using the Verify3D utility) was used to generate the Ramachandran plots and scores for all the structures modelled using I-Tasser. RMSD scores between the atoms of the guided shadow and the query protein are calculated through in-house scripts written in Python and R, developed at the Tangney lab. Amino acid sequences and/or PDB files were given as inputs wherever necessary.

### Programming

Scripts for processing the *in silico* parameter data from the servers and integrating into the Unity environment were written in Python and C# programming language. Individual scripts used in integrating the data from various servers and scripts used for calculating certain *in silico* parameters such as the RMSD score are documented in GitHub^1^.

### Graphics and Game Mechanics

Blender v2.8 was used (i) for generating all the graphical 3D files using protein structures (modelled using I-Tasser), (ii) for guided shadows, and (iii) to add flexibility to protein backbones. The Unity v2019.2.9 personal edition game engine was used for animating the opening cutscene, internal game mechanics and implementation.

## Implementation and Results

### Pepblock Builder VR as an Edutainment Tool

**FIGURE 2:**
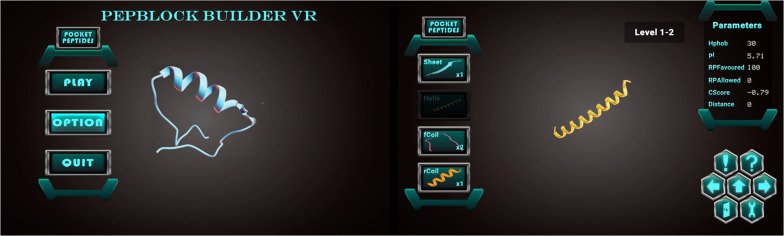
**(A)** Welcome screen of the Pepblock Builder VR showing the menu panel for play, options (sounds, music, and volume controls) and quit buttons **(B)** The game UI of the Pepblock Builder VR with the peptide panel on the left with basic secondary structures as building blocks and the right panel showing various *in silico* parameters related to the structure. The hexagonal control panel contains functions for play instructions (! symbol), undo and redo (right and left arrows), help button (! symbol), submit button (up arrow), exit button (with door symbol) and a settings toggle button.

**FIGURE 3:**
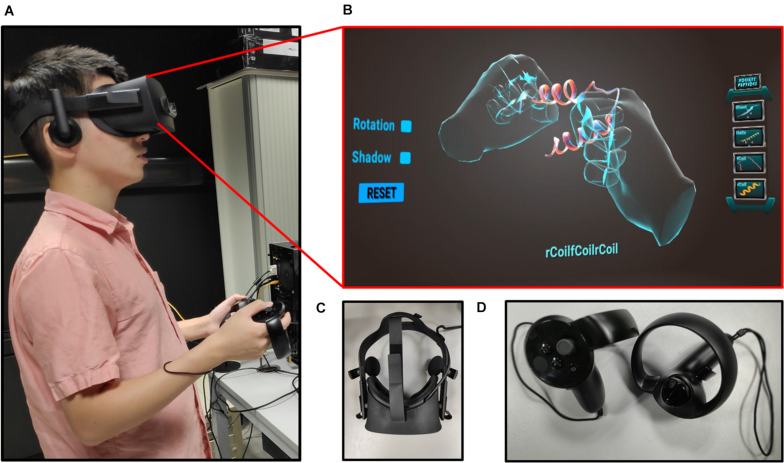
**(A)** User with Oculus Rift headset and controllers, experiencing the Pepblock Builder VR in VR. **(B)** UI of the Pepblock Builder VR VR. **(C)** Oculus rift headset used for the Pepblock Builder VR. **(D)** Handheld controllers used for user–computer interaction.

The Unity 3D game engine was used to develop the Pepblock Builder VR’s gameplay and graphics. Two versions of the Pepblock Builder VR were developed, i.e., (i) Pepblock Builder VR desktop version and (ii) Pepblock Builder VR VR version. Figure 2 shows the welcome screen and game UI for the desktop version. Figure 3 shows the VR UI and Oculus Rift S VR setup. Neon blue and Neon green colour scheme was used throughout the UI to give a space Sci-Fi effect.

### Storyboard and Game Narrative

**FIGURE 4:**
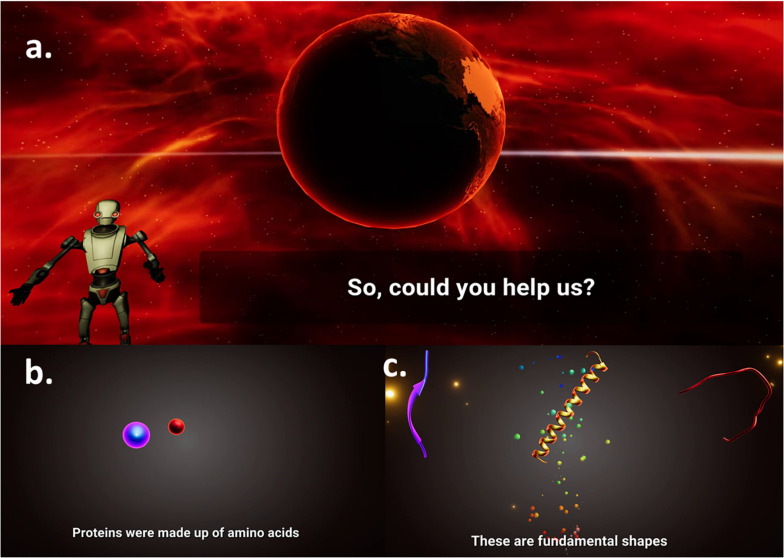
Screenshots from storyboard of the Pepblock Builder VR (Cutscene). The Pepblock Builder VR is set up in a post-apocalyptic world. **(a)** An AI guides a tutorial, talking about the grand ecosystem that existed and is now seeking help to rebuild the world. **(b)** Concepts on the composition of proteins are explained in an animated fashion. **(c)** Secondary structures of proteins (Helices, Coils, and Sheets) are projected as fundamental building blocks (LEGO style).

Modern gaming benefits from the inclusion of a captivating storyboard to narrate the game world and to introduce in-game rules to the players. The Pepblock Builder VR has a passive narrative of a human-destroyed earth and the grand ecosystem that was lost due to human activities. An AI bot seeks help for building new protein structures using three fundamental secondary shapes, i.e., Helix, Coil, and Sheet. The 60-s opening cutscene shows an animated post-apocalyptic tutorial and guides the user into building new proteins to restore life on earth. Between each task and level, an in-game screensaver presents facts about proteins displayed in curated graphics to deepen the user’s understanding of proteins. Figure 4 shows screenshots from the opening cutscene of the Pepblock Builder VR.

### Interface, Gameplay and Functionalities

The Pepblock Builder VR gameplay is based on simple LEGO style modular protein building. Users are engaged in creating novel structures based on the challenges provided by each level. The Pepblock Builder VR is designed with a progressive level-up approach to guide, teach and challenge the users toward complicated design problems using proteins.

**FIGURE 5:**
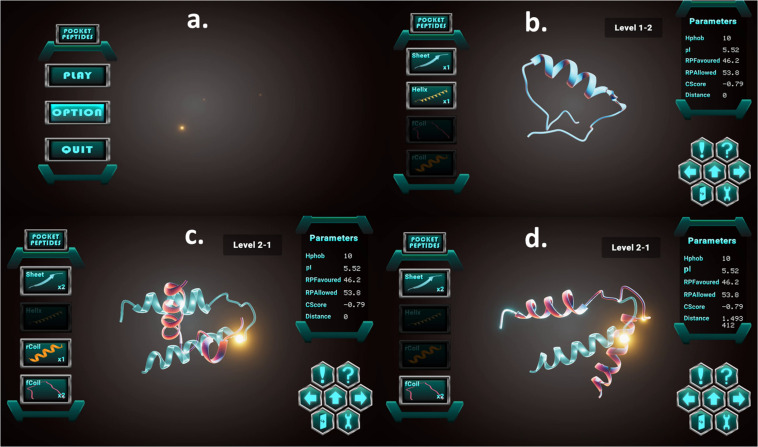
Screenshots showing the game interface and toggle buttons: **(a)** Menu board of the Pepblock Builder VR game. **(b)** A 3D visualisation of an in-game peptide, with *in silico* parameters on the right panel. **(c)** In-game challenge displaying the required final output in neon blue. **(d)** Structure manipulation by bending and snap-fitting into the required final output.

Initially (see Figure 5), the users are provided with a blank canvas in the centre, where any structure from the left panel could be dragged and dropped. The left panel has four basic shapes. The panel on the right displays the *in silico* parameters of the current structure in display. The control panel at the bottom right has a help button to provide a tour of the interface and a challenge button to display the “challenge” of the current level. The left and right arrows provide undo and redo options, respectively, while the centre up button submits the user response.

Game users are required to construct the given shape (neon blue shadow) using the basic shapes from the left panel. Subsequent levels include the ability to modify the structure by rotating, twisting and bending the protein backbone. A haptic-snaplock feature locks the protein into the shadow when the complete resemblance is achieved. For the PC version, users are allowed to click on buttons in the UI to either navigate through the various game scenes (menus and different levels) or toggle protein components (secondary structure) and tips. The protein components (displayed as icons on the left in the game scenes) are available to be dragged and dropped on the glowing area (the centre of the screen). The users are also allowed to rotate and zoom the whole scene by clicking the middle mouse button. In advanced levels, users also unlock bending, twisting and turning features by clicking and dragging on any point on the 3D structure.

The progressive level approach of the Pepblock Builder VR increases the complexity of the structures and challenges with increasing difficulties. The values in the *in silico* parameter panel, relevant to the structure displayed, change as the user modifies the structure. This promotes the basic understanding of the relationship between the *in silico* parameters and the structure of a protein. There is potential to implement more advanced levels where the challenges would demand direct structure design for a defined set of *in silico* parameters.

### Pepblock Builder VR in Virtual Reality

Although the game storyboard, narrative and game goals remain the same in VR as were in the desktop game, the user interface and user interaction were reprogrammed. The “drag and drop” feature was changed to grab and throw, which is a common user interaction method in various VR games. Controller buttons and actions for all the in-game user interactions are listed in Box 1. Users are allowed to walk around in the VR environment (available to Oculus Rift or higher). Similar to the stand-alone version, users can click on protein component icons on their left-hand side by clicking on the right trigger button on their Touch controller to display the corresponding protein model. Once a protein model is displayed at the centre of the scene, the player can grab protein models behind the protein component icons by tapping and holding the grip button on a Touch controller. After grabbing a protein model, they can examine it and be able to throw it onto the large displaying model at the centre of the scene. When a second protein model is thrown upon the existing structure, the resultant (modelled) combination of the two complexes is displayed.

**Box 1. Controls and gestures implemented for Pepblock Builder VR.**

**Table:** 

Button/action	Assigned function
Grip (L) (R)	Grab
Thumbstick (L)	Move around in VR
Thumbstick (R)	Turn around in VR
Trigger(R)	Click
Oculus (R)	Toggle Menu
Throw (action)	Merges structure in hand with the structure in display
Grip (L)/(R) + Thumbstick (L)/(R)	Moves the structure only* toward or away from the user

### Pepblock Builder VR as a Visualisation Tool

**FIGURE 6:**
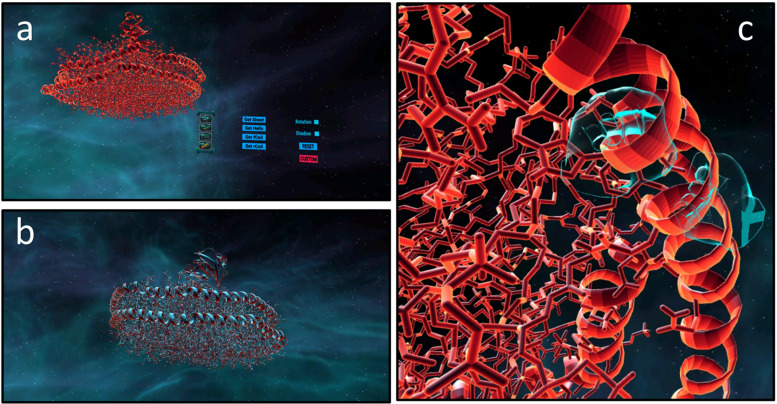
Custom protein visualisation in the Pepblock Builder VR. Protein complex “6CLZ–PDB ID” was downloaded from the RCSB PDB website and was automatically converted to a.DAE file on the Pepblock Builder VR. **(a)** Showing the VR interface for importing custom protein. **(b)** Showing 6CLZ in the Pepblock Builder VR environment. **(c)** Outer helices of the 6CLZ nano disc being rotated by virtual hands.

The gaming principles and the easy-to-navigate UI are the two key features of the Pepblock Builder VR. While the gamification forms the foundation for bio-edutainment and understanding modular protein design, the UI of the Pepblock Builder VR could be deployed to visualise any protein of interest. Any file in the.PDB format can be converted and automatically processed to be visualised in the in-game virtual environment. In the current version (the automatic processing mode), the proteins are depicted in ribbons and cartoon format by default. Other depiction models can also be achieved by manually converting any.PDB file to a.DAE file and by importing into the Pepblock Builder VR UI. An example of visualising a protein downloaded from RCSB PDB databases is shown in Figure 6.

### Pepblock Builder VR as a Protein Design Tool

The Pepblock Builder VR provides the user with a panel of LEGO-style protein building blocks. Both in the stand-alone and the VR application, the users can make combinations of these building blocks or even combine different protein structures from the PDB server. For basic experience, a certain number of permutations and combinations of the four elementary structures present in the front panel of the UI are pre-modelled and stored in the application library. Thus, when the user makes a combination of proteins using elementary shapes up to three levels, the Pepblock Builder VR instantly shows the resultant structure. However, just as the LEGO analogy, the complexities and permutations and combinations of possible structures that can be made are infinite. Thus, it would be impractical to have a resultant file for every possible protein structure made from the elementary structures. To tackle this, the Pepblock Builder VR was linked to a stand-alone protein modelling application. In the backend, the resultant structure is modelled and displayed in the Pepblock Builder VR UI when ready. In our case, I-Tasser stand-alone application was used for protein modelling. However, this is a highly time-consuming process and dramatically increases the wait times for complex structures. Once a user gets back the resultant structure, the user will be able to store the structure locally and be able to share the structure with any other user through in-game file transfer. This enables the creation of novel protein structures in a gamified and experiential manner and without the need to work with the corresponding amino acid sequences of the proteins. As a design tool, the Pepblock Builder VR shows a promising potential to bring protein design to a broader non-technical audience.

### User Response and Usability

**FIGURE 7:**
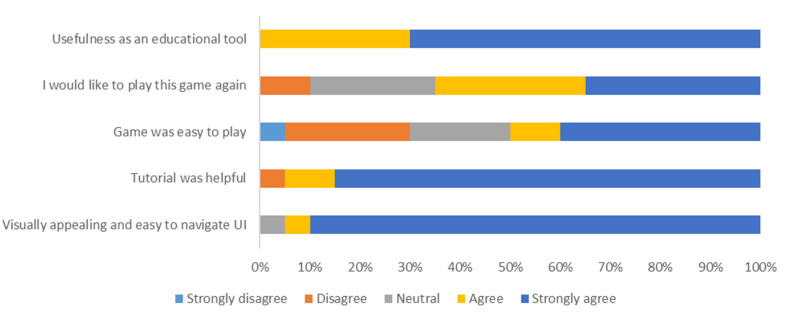
User response toward the Pepblock Builder VR. Twenty users were asked to take an anonymous survey after experiencing the game and custom protein visualisation in virtual reality.

Twenty random users with a combination of low, medium and professional knowledge on protein were asked to evaluate the Pepblock Builder VR in aspects such as usability, ease of learning about proteins, satisfaction with the VR UI and the potential for the Pepblock Builder VR as a scientific tool. The user response is shown in Figure 7.

The response shows that the vast majority of the users found the Pepblock Builder VR as a very interactive and useful bio-edutainment tool. The distribution of experiencing different levels of difficulty to play the game could be due to the varied level of prior knowledge in proteins. However, most users found the tutorial (including the cutscene) very helpful in understanding the context and the background of the game.

## Discussion and Outlook

**FIGURE 8:**
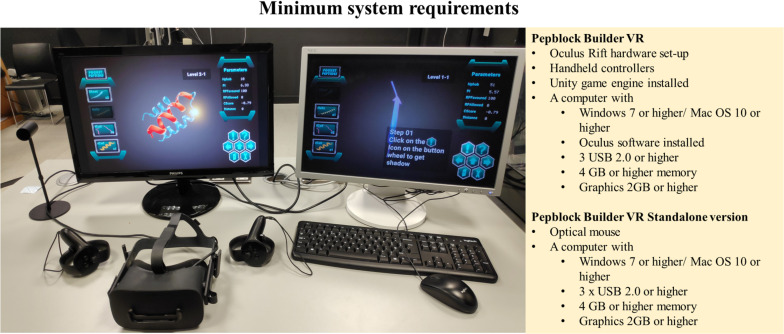
Minimum requirements for running the Pepblock Builder VR.

We developed the Pepblock Builder VR by blending the concepts of protein design, 3D visualisation and VR and exploiting the merits of gamification. Thorough care was taken during every iteration of the tool to provide an interactive interface to users of all levels. Considering that not all users would have the availability of HMD equipment for the VR environment Pepblock Builder VR package, we also packed the entire gamified learning experience into a desktop version. The minimum requirements to run the Pepblock Builder VR are described in Figure 8.

The existing VR tools for molecular visualisation are often complicated to navigate, and some tools also require basic programming skills. Compared to these, the Pepblock Builder VR has non-expert friendly, one-step installation procedure, simple navigation and workflow. The Pepblock Builder VR’s interface is designed to interact and inform the user with a guided tutorial at every screen. The cutscene and the pop-up tutorials explain the game context and provide the users with in-game help wherever required.

The Pepblock Builder VR is enriched with multiple challenges in each level and edutains the users with fun facts about proteins at the end of each level. Such a gamified learning experience is unique to the Pepblock Builder VR. Gamification principles have not been extensively used priorly in any other molecular visualisation tools except in Fold.it. The gamification of the Pepblock Builder VR adopts a LEGO-style modular protein design. Proteins are versatile biomolecules. Fusion proteins (proteins made by combining two or more full/partial proteins) and engineered versions of natural proteins have been revolutionising various fields such as biomedicine, materials technology and food processing. The concepts of protein design could be compared to a LEGO-style modular approach with a twist. For example, a combination of structures A [Helix] and B [Coil] may not be [A + B], i.e., Helix attached to a coil, and in most cases may result in a completely new structure C. This non-linear nature of combining two protein structures is difficult to understand without some basic theoretical knowledge on free energy and stereochemistry. This gets more complicated when protein design is introduced. In such cases, deploying gaming-based learning provides a solution.

Gaming principles involve core elements such as concept building, world realisation and problem solving. Users learn and adapt to the in-game principles as they progress through levels. This slow introduction of world rules and self-adapted learning of concepts is an effective alternative way compared to the traditional classroom-based learning of protein design. The process of designing solutions, modelling ideas, building strategies and testing the outputs is a form of the “design, model, build and test” approach, a cornerstone of synthetic biology (Agapakis, 2021; Bueso and Tangney, 2021). Combined with a gamified protein design interface, an immersive VR experience and a LEGO-style protein building interface, the Pepblock Builder helps in the understanding of protein folding concepts for both technical and non-technical users. This was successfully observed from the user experience survey. Nearly 90% of the users found the Pepblock Builder VR easy to navigate and visually appealing. Over 70% of the users strongly agreed that the Pepblock Builder VR is a useful educational tool.

The Pepblock Builder VR could also be used as a simple visualisation tool. Any protein structure could be visualised in VR using the Pepblock Builder VR. Protein complexes and small molecules can also be imported into the VR interface. The Pepblock Builder VR can fetch *in silico* parameter data for the imported structure by interacting with online servers and in-built scripts.

The current version of the Pepblock Builder VR is a proof-of-concept tool with several limitations. One of the key limitations of the game is the time consumed while modelling the new combinations of the protein structure. The current game database has limited permutations and combinations of the four basic shapes provided in the left panel. Levels 1–10 rely on this in-game database. As the levels progress, the demand of newer combinations will rise. This requires protein modelling. Currently, with a lab-grade computer, modelling a 100–200 amino acid chain would take 5 h and 12–15 h for 500 AA long protein, depending on the structural complexity. Real-time modelling of such structures would be challenging to achieve without long wait times. Improving the time consumed for protein modelling is a bottleneck that we aim to improve in the next iterations of the game. In addition to this, to maintain the attention of the users during the wait periods, creative subtasks and facts-based edutainment levels will be implemented.

In the current version, game levels only up to level 10 are included. We plan to introduce packages with more game levels and new challenges in the near future. The first 10 levels of the Pepblock Builder VR have an in-game shadow to direct users toward the end goal of each level. The *in silico* parameters displayed in the right panel change in real time as the protein structure is being modified. This feature becomes the primary guide for level 11 onward. The users would be challenged to make/design a structure for a defined set of *in silico* parameters. This expands the potential of the Pepblock Builder VR from a bio-edutainment and visualisation tool to a citizen-based protein design interface. In future, cloud-based libraries for user-designed proteins would be established, enabling multiplayer capabilities and providing “share and build” features.

The Pepblock Builder VR opens a new avenue in protein design. With advancements in parallel computing, increasing computational power and improving graphics, the future of bringing protein design to the masses looks promising.

## Data Availability Statement

The original contributions presented in the study are included in the article/supplementary material, further inquiries can be directed to the corresponding authors.

## Author Contributions

All authors listed have made a substantial, direct and intellectual contribution to the work, and approved it for publication.

## Conflict of Interest

The authors declare that the research was conducted in the absence of any commercial or financial relationships that could be construed as a potential conflict of interest.

## Footnotes

1 https://github.com/TIanshuXu/Pocket-Peptides-PC
